# Antibacterial effects of natural compounds on biofilm formation of S*treptococcus mutans*

**DOI:** 10.1002/cre2.673

**Published:** 2022-10-25

**Authors:** Farideh Kamarehei, Mohsen Mehdiabadi, Fariba Naderi

**Affiliations:** ^1^ Department of Microbiology, Faculty of Medicine Hamadan University of Medical Sciences Hamadan Iran; ^2^ Department of Pediatric Dentistry, Faculty of Dentistry Hamadan University of Medical Sciences Hamadan Iran

**Keywords:** antibacterial agents, biofilms, dental caries, S*treptococcus mutans*

## Abstract

*Streptococcus mutans* is the main cariogenic pathogen in the oral cavity, considered to contribute toward oral diseases. *S. mutans* is predominantly entrapped in plaque biofilms.

**Objectives:**

In this study, we surveyed the antibacterial activity of natural compounds in terms of the biofilm production of *S. mutans*.

**Material and Methods:**

We extracted the studies related to natural compounds affected on *S. mutans* biofilm from different databases.

**Results:**

Disruption of *S. mutans* viability in biofilms by a potent new pharmacological factor could inhibit and remove cavities. Various antibacterial agents are needed to destroy biofilms that remove both pathogens and commensal bacteria, and also exert inhibitory effects on many bacterial species.

**Conclusions:**

An effective therapeutic agent for dental caries has to be capable of removing pathogens and their biofilms. Specific virulence attributes of *S. mutans* exist; hence, natural compounds that have excellent properties to combat such pathogens need to be selected.

## 1 INTRODUCTION

Dental caries, one of the most infectious diseases, affect more than 4 billion people worldwide. Dental caries are one of the major infection diseases. Demineralization of the enamel can cause destruction of the tooth surface, and then, the enzymes lyse the organic compounds and dental caries are formed. Therefore, the enamel and dentin are penetrated, and the pulp is destroyed. Dental caries control measures were introduced from 1965 to 1966 (Pitts et al., 2017).

*Streptococcus mutans* is the main pathogen responsible for dental caries when an imbalance in the microbiota occurs. *S. mutans* metabolic activity, such as acid production, acid tolerance, and biofilm production, contribute to tooth decay. *S. mutans* is often isolated from plaques accompanied by the dental caries (Klein et al., 2015).

Several factors increase the pathogenicity and resistance of *S. mutans*. *S. mutans*, the main microbe that causes tooth decay, triggers imbalance using diet polysaccharide and generates different acids due to demineralization of the enamel. *S. mutans* has different pathways through which it produces carbohydrates while surviving in acidic conditions and its stress tolerance abilities contribute to dysbiosis (Klein et al., 2015).

*S. mutans* produce glucan from sucrose carbohydrate, resulting in the formation of a firm biofilm on the surface of the teeth. Then, bacterial cells attach to the matrix proteins and aggregate together. Therefore, exopolysaccharides (EPSs) form due to resistance attachment. Then, a biofilm is formed. After that, the cells communicate with each other in the mature biofilm by secreting specialized proteins and DNA. Finally, the cells disperse and form further biofilms. Anti‐biofilm agents can interrupt one or several stages of biofilm production to inhibit biofilm formation (Mishra et al., 2020).

**Figure 1:**
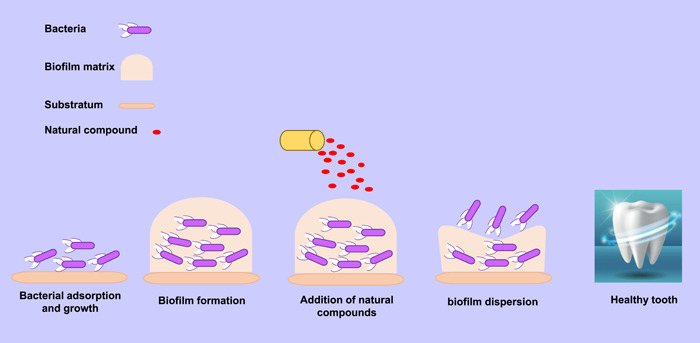
Schematic diagram of the anti‐biofilm effect of natural compounds

In this review, we have summarized the available evidence on the inhibition of *S. mutans* growth. We have focused on the origin, structure, and potential mechanism of these inhibitors. Furthermore, among small molecular agents, we have also considered antimicrobial peptides and protein inhibitors developed in this field (Figure 1).

### 1.1 Anti‐biofilm compounds against *S. mutans*

There are different classes of natural agents with high anti‐biofilm properties including phenolics, essential oils, terpenoids, lectins, alkaloids, polypeptides, and polyacetylenes (Yong et al., 2019). In this study, we have reviewed different natural compounds in comparison with other products used against biofilms of *S. mutans*.

#### 1.1.1 Commercially available compounds against *S. mutans*

Most commercially available antimicrobial agents including fluoride and chlorhexidine (CHX) are characterized by side effects and drug resistance (Kawakita et al., 2019). The synergistic effects of some materials such as trans‐cinnamaldehyde (TC) with fluoride and also CHX on *S. mutans* were examined. TC promotes the antibacterial action of CHX and fluoride and reduces *S. mutans* acid production. Also, TC could decrease *S. mutans* biofilms and alter the biofilm structure. In addition, TC could downregulate the genes related to the metabolic function, biofilm, and virulence of *S. mutans*. TC along with CHX and fluoride are not toxic to the cells and do not produce nitric oxide (Balasubramanian et al., 2021).

#### 1.1.2 Antibacterial effects of curcumin (CUR) on *S. mutans*

CUR, a natural composite of the ginger family, has been shown to have antimicrobial activity against this microorganism. Quantitative biofilm biomass and viability were assessed in a study using PCR to evaluate biofilm formation. Confocal laser scanning microscopy (CLSM) was used to assess the EPS composition, density of the biofilms, and the combination of the biofilms. The structures of the cells were also determined by scanning electron microscopy (SEM). Moreover, quantitative real‐time PCR was used to determine gene expression. The 50% minimum biofilm eradication concentration (MBEC50) of CUR against *S. mutans* was 0.5 mM. The biomass and viability reduced after the use of CUR in biofilms. Therefore, CUR could inhibit *S. mutans* biofilm production. *S. mutans* was sensitive to CUR in biofilm formation. EPS formation was decreased with the use of CUR in *S. mutans* biofilms though downregulation of glucosyltransferase enzymes and quorum‐sensing genes of *S. mutans*. Therefore, in this study, the potential inhibition effects of a natural antimicrobial, CUR, against the cavities linked to plaque biofilms were demonstrated (X. Li et al., 2019).

Recently, CUR has been recognized as a herbal therapeutic agent that is effective for use in different diseases because of its antioxidant and anti‐inflammatory properties (Marchiani et al., 2014). In a study, use of a herbal toothpaste with CUR was compared with use of a common toothpaste in patients with chronic gingivitis. It was found that herbal toothpastes with CUR were more effective than common toothpastes (Ravishankar et al., 2022). To overcome the hydrophobic nature and poor absorption of CUR, Zn2+ was added. Zn2+ facilitates smoother release of CUR from the complex. Besides, there was an improvement in osteoblast cell viability and greater osteosarcoma inhibition in the treatment group than the control group, and better antimicrobial effects  were also observed (Bhattacharjee & Bose, 2022).

Nowadays, stem cells are considered for regenerative medicine, because of proliferation and multi‐potential features (Kamarehei, 2022; Naderi et al., 2022). However, the immunomodulatory and regenerative effects of stem cells in various disorders still require to be improved. CUR, due to its anti‐inflammatory and antioxidant effects, is used for the treatment of inflammatory diseases (Ayadilord et al., 2022).

#### 1.1.3 Anti‐biofilm effects of kaempferol on *S. mutans*

Kaempferol is a natural flavonol, a kind of flavonoid, found in different plants and foods such as kale, beans, tea, spinach, and broccoli (Holland et al., 2020). Also, Quercetin found in various fruits, vegetables, leaves, seeds, and grains; red onions and kale with a bitter flavor reduce allergic responses or promote immunity. The minimum inhibitory concentration (MIC), the 50% minimum biofilm inhibition concentration (MBIC50), and the minimum biofilm reduction concentration (MBRC50) of nanovesicles derived from quercetin and kaemferol against *S. mutans* were determined. The inhibition effect on *S. mutans* biofilm production was evaluated using an in vitro biofilm model. It was found that quercetin and kaemferol could destroy biofilms in comparison with the negative control. The dry weight of biofilms, total protein, viable cells, measured by colony forming units, and glucan production were determined. The acidity of the in situ culture pH of biofilms reduced after adding quercetin and kaemferol. Quercetin and kaemferol have the capacity to destroy *S. mutans* biofilms, in comparison with CHX. Their inhibitory activities on *S. mutans* biofilms were observed in this study. Therefore, quercetin and kaemferol are important for treatment of cavities (Zeng et al., 2019).

#### 1.1.4 Anti‐biofilm effects of sphingolipids on *S. mutans*

Sphingolipids are a complex of aliphatic amino alcohols found in brain extracts and are called the mythological sphinx (Chun & Hartung, 2010). The anti‐biofilm and antibacterial effects of sphingosine, phytosphingosine (PHS), which naturally exists in skin cells, and sphinganine were examined. hydroxyapatite (HA) surfaces composed of sphingolipids with *S. mutans* were examined and the number of adhered cells was observed by cultures and a confocal microscope. The germicidal action of sphingolipids was examined using culture. Sphinganine, PHS, and sphingosine could inhibit bacteria that adhere to the surface by 1000‐, 8‐, and 5‐fold, respectively. On saliva‐coated HA, sphinganine and PHS could inhibit bacteria 10‐fold. Sphingosine had the strongest bactericidal activity in biofilms, in comparison with PHS and sphinganine. 12.5 μg/ml of PHS and sphingosine could kill the bacterial cells and disrupt the biofilm, and sphinganine could reduce the bacterial cells and disrupt the biofilm by 100‐ and 1000‐fold, respectively. Atomic force microscopy showed that mechanical stability was not related to anti‐biofilm activity. The data demonstrated that sphingolipids could be considered as an anti‐oral biofilm agent against *S. mutans* (Cukkemane et al., 2015).

#### 1.1.5 Anti‐biofilm effects of 7‐epiclusianone (7‐epi) on *S. mutans*

7‐epi is a multipurpose natural compound with potent chemotherapy, immune‐modulatory, angiogenesis‐inhibitory, and anti‐allergic features found in *Rheedia gardneriana* and *Garcinia brasiliensis*. The effects of this compound on glucosyltransferase (Gtf) B, acid production, and biofilm formation of *S. mutans* in dental caries were evaluated in vitro and in vivo. 7‐Epi could decrease the level of GtfB and also *S. mutans* biofilm formation significantly. However, it had no influence on the bacterial viability (15% ethanol, *p* > .05). In a study, 7‐epi was used topically (twice a day for 60 s) in wistar rats. It significantly reduced the number of cavity lesions, but did not reduce *S. mutans* numbers in dental biofilms. Therefore, it is clear that 7‐epi can be effective for use against dental caries in vivo and in vitro (Salles branco‐De‐Almeida et al., 2011).

#### 1.1.6 Anti‐biofilm effects of antimicrobial peptides on *S. mutans*

The antimicrobial activities of Reutericin 6 peptide, a bacteriocin produced by Lactobacillus reuteri, and gassericin A peptide, a bacteriocin generated by *Lactobacillus gasseri*, were compared with LR‐10. This study indicated that LR‐10 could affect *S. mutans* in comparison with different peptide‐based compounds. LR‐10 has a selective function against *S. mutans*. The biofilm inhibition assay demonstrated that LR‐10 exerted inhibitory effects on *S. mutans* biofilms at a low concentration (6.5 μM) in vitro. Unlike many peptides, LR‐10 disrupts the *S. mutans* cell membrane. Unlike many peptides, LR‐10 disrupts the *S. mutans* cell membrane. Besides, the hemolytic function assay and the cytotoxicity test showed that LR‐10 might be effective (Liang et al., 2019).

Moreover, the antimicrobial peptide LL‐37 (KR‐12‐3) exerted antibacterial effects on peri‐implantitis. This antimicrobial peptide, with low toxicity, had inhibitory effects on bacterial growth and biofilm formation. Also, it could reduce the production of inflammatory cytokines in stimulated cells via downregulation of the genes (Zhuo et al., 2022).

Besides, the synthetic antimicrobial peptide GH12 could downregulate the virulence genes of *S. mutans*, reduce the activity of enzymes, inactivate the regulatory system, and also inhibit EPS synthesis. Therefore, GH12 at a ½ MIC concentration could inhibit biofilm production (Wang et al., 2018).

On the other hand, the synthetic antimicrobial peptide C16G2 has a specific effect on *S. mutans* rather than other *Streptococcus species*. C16G2 disrupts the bacterial cell membrane to completely eliminate the bacteria (Kaplan et al., 2011).

Antimicrobial peptides have strong potential against pathogens, among which synthetic peptides may be more effective than natural ones, but natural compounds are regarded as safe products, without any side effects and cytotoxicity (Ageitos et al., 2017).

#### 1.1.7 Anti‐biofilm effects of antimicrobial photodynamic therapy (aPDT) on *S. mutans*

There are different therapeutic pathways to remove biofilms. In a study, the antibacterial effects of aPDT based on a visible light‐activated photosensitizer (PS) due to the production of reactive oxygen species (ROS) and removal of microbes were evaluated (Cieplik et al., 2018). aPDT by a diode laser [DL] and a chlorophyllin–phycocyanin mixture [CHL‐PC]) as a PS were used in biofilm cavity models of *S. mutans*. The lethal and subsignificant inhibitory potential of aPDT by CHL‐PC, and DL, and also the metabolic functions of *S. mutans* were examined using crystal violet and XTT reduction tests. aPDT with high levels of CHL‐PC and DL could reduce the in vitro *S. mutans* biofilm significantly. Also, CHL‐PC with aPDT reduced the metabolic function of *S. mutans*. Therefore, ROS generated in bacterial cells at high levels of CHL‐PC and DL increased significantly. Although CHL‐PC with aPDT could decrease the viability rate of *S. mutans* in biofilms and affected the metabolic functions and also the microbial virulence of *S. mutants*, CHX could destroy biofilms around 1.7‐fold in comparison with CHL‐PC with aPDT (Afrasiabi et al., 2020).

#### 1.1.8 Anti‐biofilm effects of Chilean propolis on *S. mutans*

Chilean propolis, a reddish or brown resinous substance prepared with honeybees from tree buds, is utilized to fill up gaps and seal and varnish honeycombs. Propolis is a nontoxic natural compound with different pharmacological features such as anticancer, antioxidant, antimicrobial, and anti‐inflammatory effects (Barrientos et al., 2013).

Chilean propolis has biological properties, including antibacterial and anti‐biofilm. The polyphenols in the Chilean propolis extract had an influence on *S. mutans* biofilm formation. Apigenin, quercetin, pinocembrin, and caffeic acid phenethyl ester are the main flavonoids in Chilean propolis. The polyphenol was screened in this extract using a high‐performance liquid chromatography−diode array detector. The MIC test and confocal microscopy were performed to determine the antimicrobial activity and biofilm thickness of *S. mutans*. These polyphenols, especially pinocembrin and apigenin, exerted antibacterial and anti‐biofilm effects at low concentrations against *S. mutans* (Veloz et al., 2019).

#### 1.1.9 Anti‐biofilm effects of embelin on *S. mutans*

Embelin is a para‐benzoquinone derived from berries of Embelia ribes plants. Embelin has different biological properties, such as antioxidant, antitumor, anti‐inflammatory, analgesic, anthelmintic, antifertility, and antibacterial (Radhakrishnan & Gnanamani, 2014). The embelin creates a zone of inhibition around bacterial culture, which causes periodontitis and dental caries (Kole et al., 2005).

#### 1.1.10 Anti‐biofilm effects of piperine on *S. mutans*

Piperine is an alkaloid isolated from black pepper (*Piper nigrum*), one of the most commonly comsumed condiments, and long pepper (*Piper longum*) of the Piperaceae family (Derosa et al., 2016). In a study, 30 clinical isolates were distinguished as *S. mutans* and identified for biofilm production using the microtiter plate method. Among 30 isolates identified, 18 isolates had strong biofilms, 9 isolates had moderate biofilms, and 3 isolates have poor/non‐biofilms. The isolate that produced higher numbers of biofilms (SM03) was utilized to identify MIC and MBIC. The MIC and MBIC of piperine on SM03 biofilm were lower than those of embelin. The MBIC of piperine and embelin could inhibit biofilm production of all 18 isolates (<0.05) (Dwivedi & Singh, 2016).

#### 1.1.11 Anti‐biofilm effects of stilbenoids on *S. mutans*

Stilbenoids are hydroxylated derivatives of stilbene. The structures of these compounds are C6−C2−C6. In biochemical pharmacology, they belong to the phenylpropanoid family, and most of the biosynthesis pathways are similar to those of chalcones. The majority of the stilbenoids are biosynthesized by plants, except the antihelminthic and antimicrobial stilbenoid produced from Photorhabdus luminescens (Dubrovina & Kiselev, 2017; Richardson et al., 1988; Sobolev et al., 2006; Valletta et al., 2021).

Five common stilbenoids (resveratrol, piceatannol, gnetol, pterostilbene, piceid) were examined for their antibacterial activity on *S. mutans* (UA159). The biofilm‐inhibitory concentrations of resveratrol, pterostilbene, and piceatannol were 65.2, 54.1, and 12.7 μg/ml, respectively. However, gnetol and piceid were not effective against *S. mutans* biofilms. SEM and CLSM assays indicated that 5 min after treatment with piceatannol (12.7 μg/ml), the biofilm ruptured entirely and there was a significant (one‐way ANOVA) change in the structure of the *S. mutans* biofilm formed after 48 h, without any bactericidal effect. Lactic acid proliferation was significantly reduced on using piceatannol (0.4 mmol/L), in comparison with water (0.8 mmol/L) (*p* < .05). Molecular docking analysis predicted that piceatannol can adhere to the LuxS pocket of *S. mutans*, indicating an anti‐quorum‐sensing property. Therefore, piceatannol is highly effective against *S. mutans* acid production and biofilm formation (André et al., 2017; Zhang et al., 2017).

Piceatannol and its glucoside, astringin, are phenolic agents of spruces (Bowen & Koo, 2011; Lee et al., 2001; Münzenberger et al., 1990; Yao et al., 2005). The effects of lead stilbene on GtfB and GtfC were evaluated in research by a label‐free, real‐time analysis, which showed slightly micromolar *K*D values (lower concentration) and higher affinity of the compound. Therefore, piceatannol could control growth and inhibit the biofilm production of *S. mutans* in an animal model of dental caries (Nijampatnam et al., 2018).

Resveratrol is a stilbenoid, natural phenol, and a phytoalexin produced following damage and when bacteria or fungi attack plants. Resveratrol is found in foods such as grapes, blueberries, raspberries, mulberries, and peanuts (Frémont, 2000; Jasiński et al., 2013). In spite of the fact that resveratrol is usually utilized as a nutritional supplement in animal models and human diseases, there is no notable effect on any human disease (Sahebkar et al., 2015).

The influence of resveratrol on acid production, viability rate, and EPS production (soluble and non‐soluble EPS) was measured quantitatively. Also, biofilm production by the crystal violet test and the structure were determined. Resveratrol could reduce acidogenicity, EPS, and biofilm production. Expression of virulence genes was also inactivated by increasing the resveratrol concentration (J. Li et al., 2020).

Oxyresveratrol, a stilbenoid, is found in heartwood of *Artocarpus lakoocha* and in an ancient drug “Puag‐Haad.” It is also the aglycone of mulberroside A, an agent of *Morus Alba*, the white mulberry. Oxyresveratrol is a strong tyrosinase inhibitor (Kim et al., 2010; Li et al., 2020; Maneechai et al., 2009; Park et al., 2014).

Oxyresveratrol, an agent that is present in plants, exerts an antibacterial effect on basic survival, acid production, acidurity, and EPS production of *S. mutans*. Oxyresveratrol was found to exert an inhibitory effect on the viability of *S. mutans*. Oxyresveratrol decreased the *S. mutans* viability rate at 250 μg ml‐1 and controlled the production of insoluble glucans, and also biofilm formation, by significantly reducing *gtfB* and *gtfC* gene expressions. However, the enzymatic function of lactate dehydrogenase (*ldh*) was increased by the upregulation of *ldh*. Besides, *gtfD* gene expression showed the production of soluble glucan. Also, the *vicR*, *liaR*, and *comDE* genes increasingly expressed in the cells. Therefore, oxyresveratrol can control the colonization, biofilm formation, acidogenicity, and synthesis of insoluble glucans of *S. mutans (*Wu et al., 2020 *)*.

Finally, different studies have examined the effects of extracts on the quorum‐sensing systems of various bacteria and also the prevalence of virulence genes of different bacteria (Arabestani et al., 2022; Dadgar et al., 2014; Kamarehei et al., 2020; Kavyani et al., 2019; Nouri et al., 2022; Vaziriamjad et al., 2022). The properties and the antibacterial effects of different natural compounds against *S. mutans* are presented in Table 1.

**Table 1:** The antibacterial effects of natural compounds on *S. mutans*

Natural compound	Composition	Extracted from	Inhibitory effects	Biofilm biomass	Viability Rate	EPS^a^ formation	Glucosyltransferase enzymes & quorum‐sensing genes	References
Curcumin	Curcuminoid	Ginger	MBEC50^b^ was reduced	Reduced	Reduced	Reduced	Reduced	X. Li et al. (2019)
Kaempferol	Natural flavonol	Beans	MIC^c^, MBIC50,^d^ MBRC50^e^ were reduced	Reduced	Reduced	Reduced	Reduced	Zeng et al. (2019)
Quercetin	Flavonol	Vegetables	MIC, MBIC50, MBRC50 were reduced	Reduced	Reduced	Reduced	Reduced	Zeng et al. (2019)
Sphingolipids	Aliphatic amino alcohols	Tissues	MIC was reduced	Reduced	Reduced	Reduced	Reduced	Cukkemane et al. (2015)
Flavonoids (pinocembrin, apigenin)	Polyphenols	Chilean propolis	MIC was reduced	Reduced	Reduced	Reduced	Reduced	Veloz et al. (2019)
Embelin	Para‐benzoquinone	Embelia ribes	MIC was reduced	Reduced	Reduced	Reduced	Reduced	Kole et al. (2005)
Piperine	Alkaloid	Black Pepper	MIC, MBIC were reduced	Reduced	Reduced	Reduced	Reduced	Derosa et al. (2016)
Stilbenoids (piceatannol)	Phenylpropanoids	Spruces	MIC was reduced	Reduced	Reduced	Reduced	Reduced	André et al. (2017)
Stilbenoids (resveratrol)	Phenol	Plant	MIC was reduced	Reduced	Reduced	Reduced	Reduced	Yao et al. (2005)
Stilbenoids (oxyresveratrol)	Phenol	Heartwood	MIC was reduced	Reduced	Reduced	Reduced	Reduced	Wu et al. (2020)

^a^ Exopolysaccharide (EPS).

^b^ 50% minimum biofilm eradication concentration (MBEC50).

^c^ Minimum inhibitory concentration (MIC).

^d^ 50% minimum biofilm inhibition concentration (MBIC50).

^e^ Minimum biofilm reduction concentration (MBRC50).

## 2 CONCLUSION AND OUTLOOK

*S. mutans* is a main cariogenic pathogen in the oral cavity that contributes to many oral diseases. *S. mutans* is predominantly encased in plaque biofilms. Selective omission of dental caries is the best treatment option. Anti‐biofilm agents can inhibit the growth of *S. mutans* at micro‐areas of teeth, dental restoration, or implant‐supported prostheses. However, currently, oral antimicrobial agents are utilized primarily as broad‐spectrum bactericides, and they poorly regulate the production of both biofilms and virulence factors. Oral care agents, such as CHX, and so forth, are suitable and the combinatorial effects of them were still investigating. Since the chemical agents kill both pathogens and commensal bacteria, therefore, natural agents have been considered for the preventive treatment of dental caries.

Therefore, in this study, the potential of some natural agents was investigated on the biofilm‐promoting property of *S. mutans*. These natural agents exerted considerable biological effects and could protect the teeth from the injuries caused by *S. mutans*. The influence of natural agents on *S. mutans* is linked to different pathways that reduce microbial colonization and then control biofilm formation, and may even lead to alterations in biofilm structures, for example, disruption of the LuxS quorum‐sensing system by piceatannol, downregulation of *gtfB, gtfC*, and *ldh* genes involved in EPS production by oxyresveratrol, and also reduction of acidity by quercetin and kaemferol. This study identified various natural agents with potent anti‐biofilm effects on *S. mutans*. Also, a promising anticariogenic agent against *S. mutans* was identified. Therefore, the most effective compound has to be chosen for further investigations. On the other hand, the mechanism of antimicrobial natural compounds should discover. In conclusion, disruption of *S. mutans* viability in biofilms by natural factors exerts inhibitory effects on many bacteria and prevents dental caries. Specific virulence attributes to *S. mutans* were found; hence, natural compounds with excellent properties to combat such pathogens should be selected.

## AUTHOR CONTRIBUTIONS

Farideh Kamarehei and Mohsen Mehdiabadi were involved in study conception and design. Fariba Naderi was involved in data collection. Farideh Kamarehei was involved in analysis and interpretation of results. Mohsen Mehdiabadi and Fariba Naderi were involved in draft manuscript preparation. All authors reviewed the results and approved the final version of the manuscript.

## CONFLICT OF INTEREST

The authors declare no conflict of interest.

## ETHICS STATEMENT

The present study was approved by the Ethics Committee of Hamadan University of Medical Sciences, Hamadan, Iran.

## Data Availability

The data that support the findings of this study are available from the corresponding author upon reasonable request.
